# Insight to Microbial Fe(III) Reduction Mediated by Redox-Active Humic Acids with Varied Redox Potentials

**DOI:** 10.3390/ijerph18136807

**Published:** 2021-06-24

**Authors:** Jingtao Duan, Zhiyuan Xu, Zhen Yang, Jie Jiang

**Affiliations:** 1 College of Environmental Science and Engineering, Beijing Forestry University, Beijing 100083, China; duanjingtao116@bjfu.edu.cn (J.D.); zhiyuanxu13@gmail.com (Z.X.); 2 College of Urban and Environmental Science, Peking University, Beijing 100871, China; zhen.yang-urban@pku.edu.cn

**Keywords:** low molecular weight fraction humic acids (LMWF HA), electrochemical reduction, microbial Fe(III) reduction, redox potentials (E_h_)

## Abstract

Redox-active humic acids (HA) are ubiquitous in terrestrial and aquatic systems and are involved in numerous electron transfer reactions affecting biogeochemical processes and fates of pollutants in soil environments. Redox-active contaminants are trapped in soil micropores (<2 nm) that have limited access to microbes and HA. Therefore, the contaminants whose molecular structure and properties are not damaged accumulate in the soil micropores and become potential pollution sources. Electron transfer capacities (ETC) of HA reflecting redox activities of low molecular weight fraction (LMWF, <2.5) HA can be detected by an electrochemical method, which is related to redox potentials (E_h_) in soil and aquatic environments. Nevertheless, electron accepting capacities (EAC) and electron donating capacities (EDC) of these LMWF HA at different E_h_ are still unknown. EDC and EAC of different molecular weight HA at different E_h_ were analyzed using electrochemical methods. EAC of LMWF at −0.59 V was 12 times higher than that at −0.49 V, while EAC increased to 2.6 times when the Eh decreased from −0.59 V to −0.69 V. Afterward, LMWF can act as a shuttle to stimulate microbial Fe(III) reduction processes in microbial reduction experiments. Additionally, EAC by electrochemical analysis at a range of −0.49–−0.59 V was comparable to total calculated ETC of different molecular weight fractions of HA by microbial reduction. Therefore, it is indicated that redox-active functional groups that can be reduced at E_h_ range of −0.49–−0.59 are available to microbial reduction. This finding contributes to a novel perspective in the protection and remediation of the groundwater environment in the biogeochemistry process.

## 1. Introduction

Humic acids (HA) as redox-active organic compounds can directly or indirectly affect the fates of pollutants in soil and water environments [1,2,3,4]. HA can reduce Mn(IV) and Hg(II) via serving as an electron donor in soils and sediments and thus play a role in the abiotic dark reduction in waters, sediments, and soils [4,5,6]. HA are supramolecular compounds of diverse self-assembled low molecular mass organic molecules, which form dynamic associations linked through hydrogen bridges and hydrophobic bonds [7,8,9]. Low molecular weight fraction (LMWF) of HA can be isolated by a new membrane dialysis method, and they can flow into soil micropores where many redox-active pollutants are trapped [10]. LMWF HA provides the possibility of bioremediation to treat active-redox contaminants in soil micropores.

Redox activities of HA are associated with the number of redox-active functional groups (RAFGs) and redox potentials (E_h_). HA possess electron accepting capacities (EAC) and electron donating capacities (EDC) depending on the state of RAFGs [1,11,12] including electron donating groups [13,14,15] and electron accepting groups [16,17,18]. Studies have shown that RAFGs of humus varies at different E_h_. At pH 7, the distribution range of E_h_ is −200–+300 mV [19]. Many studies have expanded the range of oxidation state potential distribution from +320 mV to +700 mV [20], and to +778 mV (pH = 5) [21]. Peat soil HA can be reduced and oxidized in a wide varied E_h_ range of −0.9 to +1.0 V by electrochemical methods [2]. By using direct electrochemical reduction/oxidation of HA at a glassy carbon working electrode, the monitored time–current curve at different E_h_ can be obtained and the EAC and EDC can be accurately calculated [1]. Although strong redox capacities of LMWF HA have been demonstrated by chemical reduction analysis [10], it has not been clarified the availability of E_h_ range at which that EAC and EDC of LMWF HA can be detected, and to what extent of EDC of LMWF HA at specific E_h_ by this novel electrochemical method.

Ferrous iron compounds/minerals are able to function as reductants for organic and inorganic contaminants including nitro benzenes, arsenic, and selenium [1,22,23,24]. Fe(III)-oxyhydroxides and Fe(III)-bearing clay minerals (e.g., ferrihydrite (Fh)) predominantly occurs as poorly soluble forms in natural environments [25]. Thus, the microbially mediated iron reduction can only be achieved by transferring electrons extracellularly as insoluble Fe(III) minerals cannot diffuse into the interior of the dissimilatory iron-reducing prokaryotes (e.g., *Shewanella* spp.). The extracellular electron transfer (EET) pathways, including HA function as an electron shuttle, are developed to mediate electrons’ transfer between microbes and poorly soluble Fe(III) minerals [26,27,28]. Microbes can donate electrons to native HA and then reduce HA flow into and/or out of the soil micropores to change the fate of redox-active minerals (i.e., iron) or contaminants [29,30]. Electron shuttle capacities (ESC) of HA [2,31,32,33] and the microbial EET pathways are highly dependent on cell membrane potential [34,35,36,37]. Until now, whether LMWF HA could act as an electron shuttle to stimulate microbial Fe(III) reduction under available E_h_ range, and the differences and link of ETC between electrochemical and microbial reduction has not been fully studied.

To this end, we collected different molecular weight fractions (LMWF and retentate) using a dialysis method, with the pore size diameter of 1.25 nm and 2.5 nm as in the previous study [10]. Subsequently, the aims of this study were (i) to determine electron exchange capacity (EEC), including electron EAC and EDC of LMWF, retentate, and bulk HA at a varied E_h_ range of −0.49 to −0.69 V using electrochemical analysis; (ii) to determine ESC of LMWF, retentate, and bulk HA using microbial Fh reduction experiments in comparison to EEC by electrochemical analysis; and (iii) to develop a potential relationship between EEC by electrochemical method and ESC by microbial method. This study aims to provide a better understanding of the electron transfer process of HA during the biogeochemistry process thus predicting the potential fates of pollutants in soil and groundwater environments.

## 2. Materials and Methods

### 2.1. Preparation of HA Solution

0.25 g Leonardite humic acids Standard (LHA) and 0.25 g Pahokee peat humic acids Standard (PPHA), purchased from the International Humic Substances Society (IHSS), were dissolved with 50 mM phosphate buffer solution (PP buffer, pH = 7) respectively and reached a final concentration of 0.5 mg/mL. The HA samples were stored in blue-capped bottles covered with aluminum foil wrap and then stored in the fridge (4 °C) to inhibit the photochemical reaction.

### 2.2. Dialysis Experiment

Dialysis bags were purchased from MWCO Regenerate (VISKASE, US). MD34-3500 and MD34-14,000 with a pore size of 1.25 nm and 2.5 nm were chosen in this study. Cut out dialysis bags of a certain length were soaked in deionized water for 24 h before dialysis experiments to remove impurities from itself effectively. Fifty milliliters of bulk HA (0.5 mg/mL) was transferred accurately into the pretreated dialysis bag. After being sealed with dialysis clamps, it was put into a 400 mL beaker containing 200 mL buffer and then totally immersed. Before being put on a magnetic stirrer (IKA, Germany) constantly stirring (500 rpm) for 72 h, the beaker was sealed with a sealing film to avoid debris from entering and the whole device was wrapped with aluminum foil wrap to prevent a photochemical reaction. The molecular size that less than 3500 (or 14,000) dalton HA fractions would release into the solvent (PP buffer) during the dialysis process, which were named as LMWF, and those fractions that failed to be out of the dialysis bag were named as retentate. Total organic carbon (TOC) contents of bulk LHA, LMWF HA, and retentate HA were determined respectively by a TOC-Vcsn analyzer (SHIMADZU).

### 2.3. Electrochemical Analysis with Direct Electrochemical Reduction and Oxidation

All electrochemical experiments were conducted in an anoxic glove box (100% N_2_ atmosphere). A platinum disk working electrode (CHI102), a Pt wire counter electrode (CHI115), and an Ag/AgCl reference electrode (CHI111) (CH Instruments, Inc., Austin, TX, USA) composed of the electrolytic cell system. The current response I was determined by CH Instruments 660E (Austin, TX, USA). The supporting electrolyte was 0.1 M KCl during the electrochemical experiment. Each sample was direct electrochemical reduction at various electrode potentials of −0.49 V, −0.59 V, and −0.69 V, respectively, and was direct electrochemical oxidation at +0.61 V. EDC, EAC, and EEC (mmole-/g C) of different molecular weight HA samples were calculated and normalized to TOC as previously described by Xu et al. [12] as follow:

(1) $$\mathrm{EDC}=\frac{Q_{\mathrm{DEO}}}{\mathrm{TOC}\times V}=\frac{\int \frac{I_{\mathrm{OX}}}{F}\mathrm{dt}}{\mathrm{TOC}\times V}$$

(2) $$\mathrm{EAC}=\frac{Q_{\mathrm{DER}}}{\mathrm{TOC}\times V}=\frac{\int \frac{I_{\mathrm{RE}}}{F}\mathrm{dt}}{\mathrm{TOC}\times V}$$

Q_DER_ and Q_DEO_ (μmol) were the number of transferred electrons in direct electrochemical reduction/oxidation experiments, F is a Faraday constant (96,485 (C/mol)) and V(mL) is a volume of reaction samples.

### 2.4. Microbiological Analysis with *Shewanella oneidensis* MR-1 Fe(III) Reduction

Experiments were carried out under anaerobic conditions in the glove box (pH 7, 100% N_2_). The total volume of the reactor was 2 mL, in which the volume of *Shewanella oneidensis* MR-1 (OD = 1) was 200 μL, the concentration of Fh (the terminal electron acceptor) was 1 mmol/L, the volume of LMWF HA/retentate or bulk was 1000/100 μL, and the remaining part was filled up with *Shewanella* basal medium SBM containing lactate (the sole electron donor). Fh was prepared according to Cornell and Schwertmann [38], and the amount of Fe(II) was quantified as previously described by Stookey [39].

ESC is defined as the stimulation effect of HA on microbial Fe(III) reduction process. Numerically ESC is equal to the difference in the amount of Fe(II) (μmol) produced in the presence and absence (blank experiment) of HA samples in microbial Fe(III) reduction experiments. Considering different molecular weight fractions have different TOC contents, ESC (mmol e-/g C) of each sample was normalized to TOC.

## 3. Results and Discussion

### 3.1. Dialysis Experiment and TOC Contents

High-performance size-exclusion chromatography (HPSEC) is used in the past decades to isolate different molecular weights of HA [40], while we select the dialysis method to collect different molecular weight fractions of HA to better compare to the results of our previous studies. In this study, dialysis experiments were conducted within 3 days (72 h) to obtain different molecular weight HA fractions. The TOC contents of all samples were shown in Table 1. The results showed diversity in TOC between LMWF and bulk HA. 3500-LMWF of LHA and PPHA account for 2.0% and 3.6% of bulk HA, respectively, which were comparable to our previous study [10]. In contrast, retentate HA of all samples had similar TOC contents with the bulk HA. TOC contents of LMWF PPHA and bulk PPHA were higher than those of LHA, indicating that bulk PPHA was able to release more LMWF compared to bulk LHA during dialysis. TOC contents of 14,000-LMWF LHA (9.14 mg C/L) and 14,000-LMWF PPHA (11.62 mg C/L) were higher than 3500-LMWF LHA (4.61 mg C/L) and 3500-LMWF PPHA (8.48 mg C/L) (Table 1), which demonstrated that LMWF ranged from 3500–14,000 Dalton has a contribution to bulk HA TOC contents. In addition, the TOC of 3500-LMWF and 14,000-LMWF HA are in the range of concentrations that are common in terrestrial and aquatic environments including rivers, oceans, estuaries, and sediments [41,42,43,44,45,46] where the concentration of dissolved organic carbon ranges from 7 to 30 mg C/L in nature [47,48].

### 3.2. Direct Electrochemical Reduction/Oxidation of Different Molecular Weight Fractions of HA Samples

**Figure 1:**
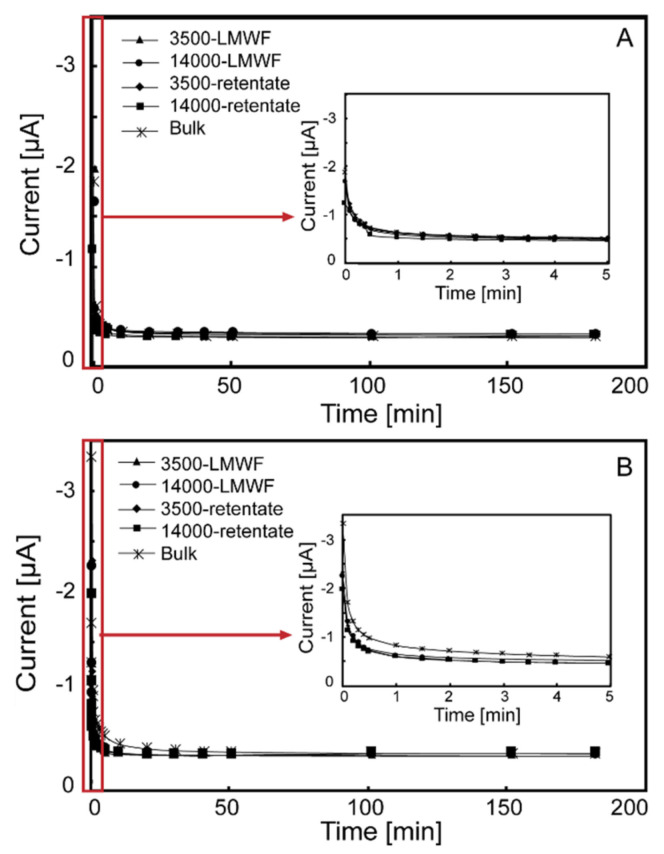
The I–t curve of different molecular weight fractions of HA in direct electrochemical oxidation experiments (The embedded picture is the I–t curve within 3 min of reaction.) (**A**) LHA; (**B**) PPHA.

**Figure 2:**
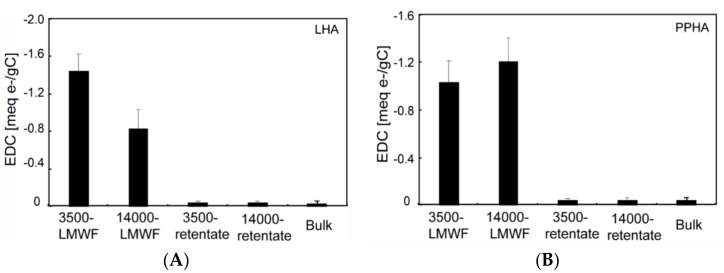
The EDC of different molecular weight fractions of LHA under +0.61 V direct electrochemical oxidation experiments. (**A**) LHA; (**B**) PPHA.

Electrochemical methods were used to investigate the contribution of LMWF, retentate, and bulk HA to EEC which is the sum of EAC and EDC. Direct electrochemical reduction/oxidation were used to monitor current I over the time t (s) of different molecules HA in E_h_ range of −0.69 V to +0.61 V by electrochemical analysis in this study. Direct electrochemical oxidation experiments were conducted at +0.61 V. Current I–t of LMWF, retentate, and bulk HA are showed in Figure 1. EDC at +0.61 V were calculated based on oxidative current I–t and were normalized to TOC contents (Figure 2). EDC was detected for all LMWF HA with −0.8 to −1.4 meq e^−^/g C, and retentate and bulk HA with −0.03 to −0.04 meq e^−^/g C. This result suggested that different molecular weight fractions of LHA and PPHA had different EDC. Compared to LMWF HA, retentate and bulk HA showed a lower EDC, which means LMWF is the main contributor in donating electrons.

**Figure 3:**
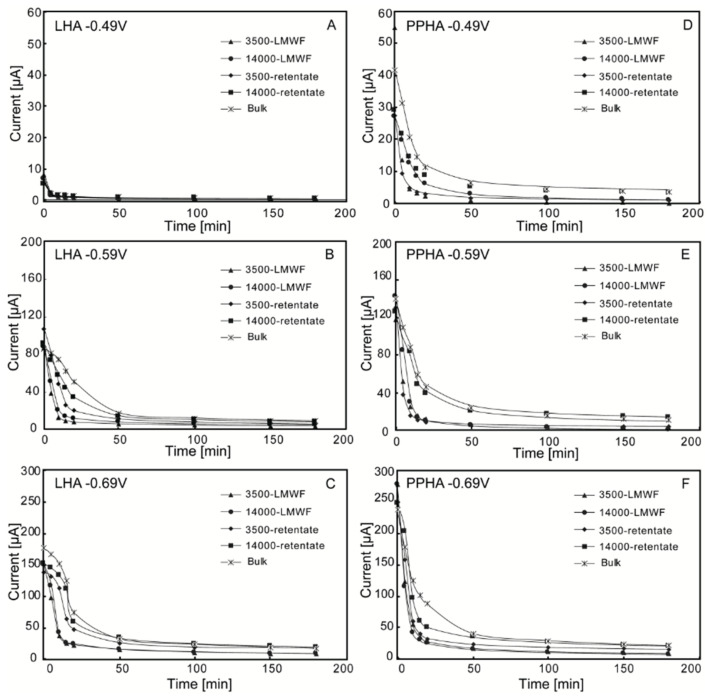
The I–t curve of different molecular weight fractions of LHA and PPHA in direct electrochemical reduction, which was carried out at Eh = −0.49 V, Eh = −0.59 V, and Eh = −0.69 V for 3 h: (**A**) LHA −0.49 V; (**B**) LHA −0.59 V; (**C**) LHA −0.69 V; (**D**) PPHA −0.49 V; (**E**) PPHA −0.59 V; (**F**) PPHA −0.69 V.

**Figure 4:**
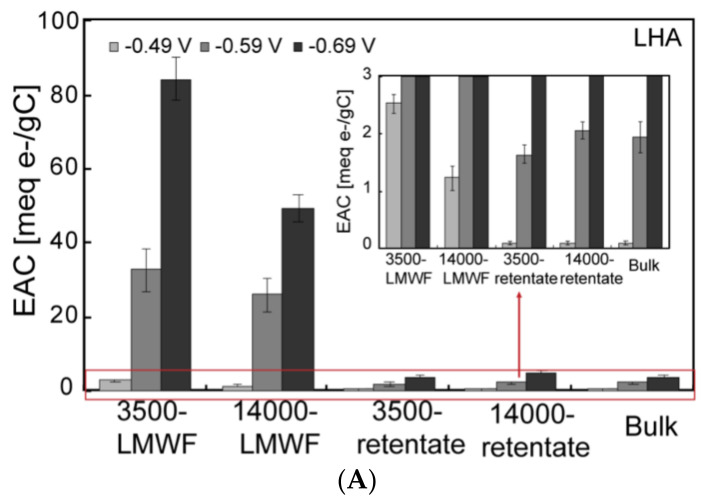

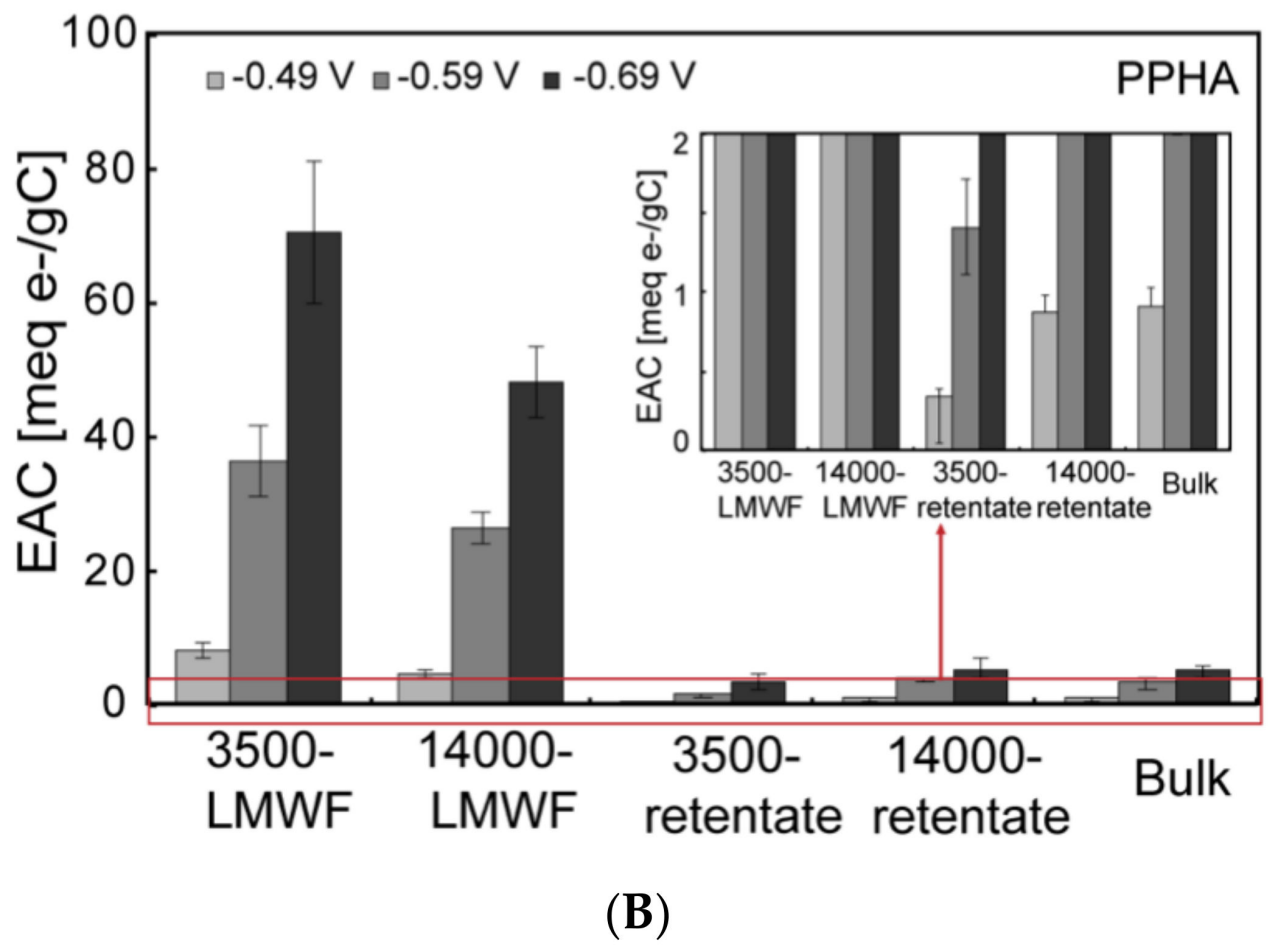
The EAC of different molecular weight fractions of HA under E_h_ = −0.49 V, E_h_ = −0.59 V, and E_h_ = −0.69 V direct electrochemical reduction experiments. (**A**) LHA; (**B**) PPHA.

Direct electrochemical reduction experiments were conducted under three E_h_ (−0.49 V; −0.59 V; −0.69 V). Current I–t and corresponding EAC are showed in Figure 3 and Figure 4, respectively. Current I–t results indicated a similar decreasing tendency of PPHA and LHA. In addition, the current I–t of all molecular fractions of both PPHA and LHA increased with a decreasing E_h_ from −0.49 V to −0.69 V, revealing that EAC of HA was highly related to environment E_h_. Additionally, EAC at −0.59 V was 12 times more than that at −0.49 V, while EAC increased 2.6 times when the potential decreased from −0.59 V to −0.69 V. These results further declared that although EAC of HA was dependent on the E_h_ of the environment, the significant enhancement of EAC between −0.49 V and −0.59 V illustrated that there were many RAFGs in HA within this range of potential. EAC of different molecular weight fractions of PPHA and LHA increased with E_h_ decreasing. LMWF showed a remarkably higher EAC per gram of carbon compared to retentate and bulk HA, which was consistent with previous studies [10], indicating that LMWF had a larger number of RAFGs than bulk HA.

Direct electrochemical reduction and oxidation experiments (Figure 1 and Figure 3) and corresponding EDC and EAC (Figure 2 and Figure 4) of all different molecular weight LHA and PPHA illustrated that HA was able to act as redox buffers, which are in agreement with previous studies [2,49]. EAC and EDC of different molecular weight fractions had been normalized to the number of electrons transferred per gram carbon (meq e-/g C) to further compare EAC and EDC of different molecular weight LHA and PPHA. Results showed that EDC of LHA and PPHA were significantly smaller than their EAC (Figure 2 and Figure 4), indicating that native HA were easier to be reduced than to be oxidized.

### 3.3. ESC of Different Molecular Weight Fractions of HA Samples

**Figure 5:**
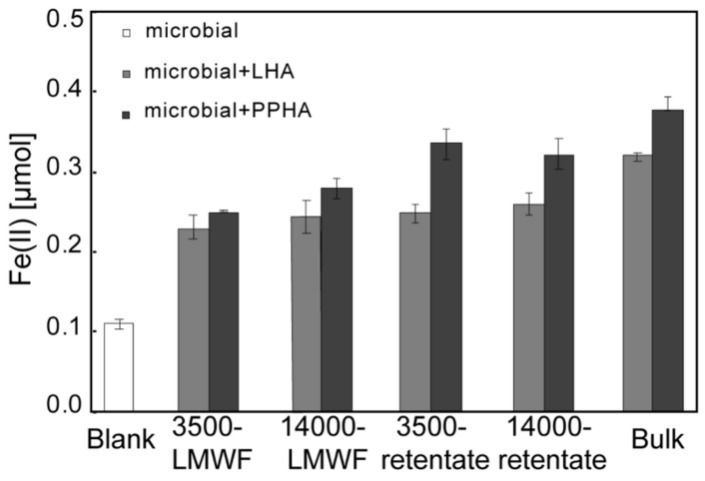
Amount of Fe (II) formation in the microbial (*Shewanella oneidensis* MR-1) Fh reduction process in the presence and absence of different molecular weight fractions of LHA and PPHA.

LMWF HA showed a strong EAC by electrochemical reduction; however, whether LMWF HA can act as an electron shuttle to stimulate electron transfer in the microbial Fh reduction process is not clear. Therefore, microbial Fe(III) reduction experiments were conducted in the presence and absence of different molecular weight fractions of HA to clarify the stimulation effect. In the 2 mL reactor, LMWF, retentate, and bulk HA were reduced by *Shewanella oneidensis* MR-1, respectively, and then they could act as electron shuttles to accelerate microbial-Fh reduction processes. There was less Fe(II) formation in the presence of LMWF HA compared to those with retentate and bulk (Figure 5), indicating that the redox capabilities of different molecular weight fractions of HA were highly dependent on its TOC contents, which was consistent with our electrochemical results. Additionally, there was more Fe(II) formation when Fh was reduced by the microbial reduced HA compared to directly reduced by *Shewanella oneidensis* MR-1 (Figure 5), showing that all different molecular weight fraction HA can act as an electron shuttle to stimulate microbial-Fh reduction processes.

**Figure 6:**
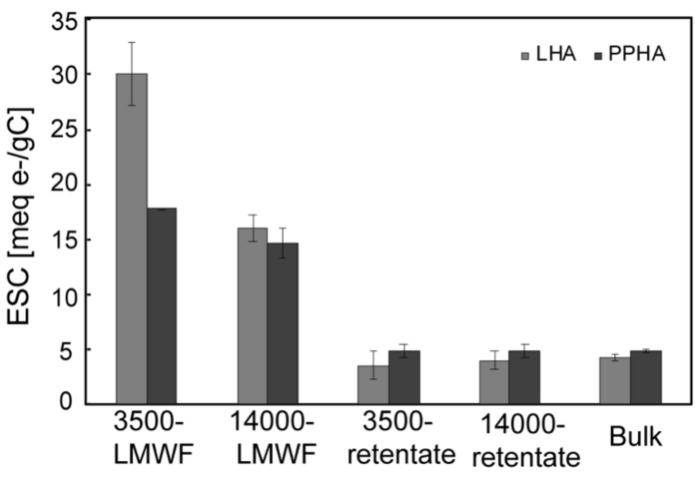
Comparison of electron shuttle capability between LHA and PPHA with different molecular weight.

ESC of different molecular weight HA was normalized to its TOC contents (meq e-/g C) (Figure 6). LMWF LHA and PPHA were 3.8–8.5 and 3.0–3.7 times higher than that of the retentate and bulk LHA and PPHA respectively, which was in accordance with electrochemical results describing high EAC of LMWF HA. It has been proved that bulk HA can facilitate the reduction of iron(III) oxide in sediments and soils [50]. Our results further demonstrate that LMWF with high EAC can also act as an electron shuttle in the microbial Fe(III) reduction process. Additionally, microbial reduced LMWF HA maybe serve as a crucial electron shuttle to reduce pollutants (Hg(II), U(VI), and Cr(VI)) and convert them to less toxic forms or remove them [51], when those contaminates are occluded in tight pore spaces that microorganisms cannot enter, particularly.

**Figure 7:**
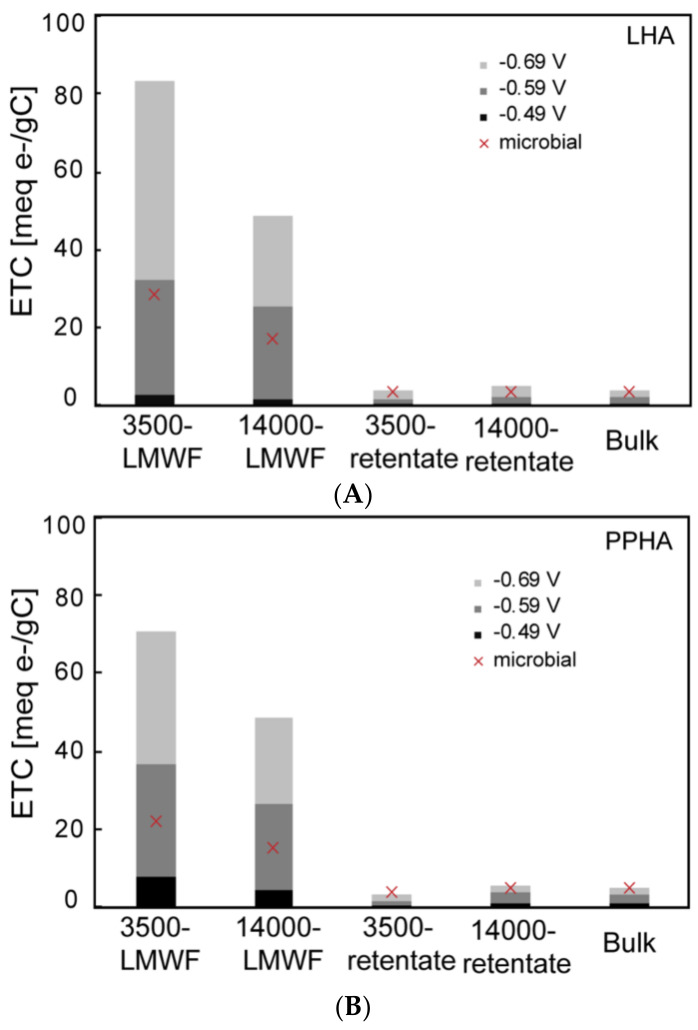
Comparison in per gram carbon ETC between electrochemical reduction and microbial reduction of HA with different molecular weight fractions. (**A**) LHA (**B**) PPHA.

EAC and ESC revealed electron transfer capacities (ETC) in different conditions. EAC reflected redox capability of different molecular weight HA at 3 specifics E_h_ values and ESC showed their capabilities in a changing E_h_ environment. HA may be reduced under varying redox conditions and the potential ranges of microbial reduction is −0.60 to 0.24 V [52]. In our study, the membrane potential of *Shewanella oneidensis* MR-1 would keep changing since the whole process is a dynamic system of continuous Fe(II) formation, which would then have an impact on the electron transfer both from microbial to HA and HA to Fh. For further comparison ETC of different molecular weight HA reduced electrochemically and microbially, ETC in the 2 mL reactors was calculated and showed in Table 2, per gram of carbon ETC are showed in Figure 7. The electron-carrying capacities of PPHA and LHA measured in an anaerobic chamber [53] were 0.87 meq/g C and 0.78 meq/g C, respectively, which are comparable with ETC of bulk PPHA and LHA in our study.

While PPHA showed a higher ETC than LHA when in 4 mL reactors (Table 2), LHA showed a higher ETC than PPHA when considering the effect of TOC, demonstrating that TOC had a significant influence when calculating ETC. Table 2 and Figure 7 showed that ETC of different molecular weight fractions of PPHA and LHA by microbial reduction was comparable to ETC by electrochemical analysis at a range of −0.49–−0.59 V whether considering per unit volume or per TOC, which suggests that an EET pathway can be achieved between Fe(III)-reducing bacteria (*S. oneidensis* MR-1) and native different molecular weight fractions of PPHA and LHA in the E_h_ range of −0.49–−0.59 V and this is comparable to the previous study [52].

## 4. Conclusions

Redox properties of HA, which are further pertinent to the fate of transformation and transport of redox-active contaminants in the environment, are highly dependent on the chemical composition and characteristics of HA such as E_h_ and RAFGs. The present study, for the first time, investigated the difference in ETC of LMWF, retentate, and bulk LHA and PPHA between microbial and electrochemical reduction. The results indicated that EAC of all molecular weight LHA and PPHA increased with a reduced E_h_ range from −0.49 V to −0.69 V. LMWF LHA and PPHA showed dramatic higher EAC compared to retentate and bulk LHA and PPHA. Microbial reduced LMWF LHA and PPHA also showed strong ESC, and their ETC was comparable to that reduced electrochemically in the E_h_ range of −0.49 to −0.59 V, which further illustrated that LMWF LHA and PPHA could act as an electron shuttle between *Shewanella oneidensis* MR-1 and Fh in this E_h_ condition. Our study provides a new perspective in the electron transfer processes via directed towards microbial and electrochemical research to identify redox-active HS in varied E_h_ soil conditions, which may link to predicate degradation and redox of pollutants trapped in soil micropores (<2 nm). LMWF HA can provide the direct or indirect interactions with pollutants (POPs, As, P, Hg, U, and Cr) trapped in soil micropore and then change the fate of the contaminate involved in microbial Fe(III) reduction processes.

## Figures and Tables

**Table 1:** TOC contents of different molecular weight fractions of LHA and PPHA after 72 h dialysis.

TOC (mg C/L)	3500-LMWF	14,000-LMWF	3500-Retentate	14,000-Retentate	Bulk	3500-LMWF/Bulk	14,000-LMWF/Bulk
PPHA	8.48	11.62	210.90	198.70	234.90	3.61%	4.95%
LHA	4.61	9.14	217.18	195.74	228.00	2.02%	4.01%

**Table 2:** Comparison in ETC between electrochemical reduction and microbial reduction of HA with different molecular weight fractions.

ETC (μmol e-)		Electrochemical Reduced			Microbial Reduced
		−0.49 V	−0.59 V	−0.69 V	
PPHA	3500-LMWF	0.14	0.62	1.20	0.60
	14,000-LMWF	0.11	0.62	1.12	0.68
	3500 retentate	0.15	0.59	1.43	0.82
	14,000 retentate	0.35	1.53	2.14	0.78
	bulk	0.43	1.57	2.33	0.92
LHA	3500-LMWF	0.03	0.40	1.04	0.56
	14,000-LMWF	0.03	0.54	1.03	0.58
	3500 retentate	0.04	0.79	1.76	0.62
	14,000 retentate	0.05	0.96	2.17	0.62
	bulk	0.06	1.14	2.16	0.78

## Data Availability

The experimental data used to support the findings of this study are included in the article. And more detailed data are available from the corresponding author upon request.
